# Risk factors for suicide behaviors in the observational schizophrenia outpatient health outcomes (SOHO) study

**DOI:** 10.1186/1471-244X-12-83

**Published:** 2012-07-19

**Authors:** Roberto Brugnoli, Diego Novick, Josep Maria Haro, Andrea Rossi, Marco Bortolomasi, Sonia Frediani, Giuseppe Borgherini

**Affiliations:** 1 Italian Foundation for the study of Schizophrenia, Rome, Italy; 2 Eli Lilly and Company, Windlesham, Surrey, UK; 3 Parc Sanitari Sant Joan de Déu, CIBERSAM, Sant Boi de Llobregat, Barcelona, Spain; 4 Medical Department, Eli Lilly, Florence, Italy; 5 Psychiatric Dept. “Villa S. Chiara” Nursing home, Verona, Italy; 6 Affective Disorders Unit, Lime Tree Park Nursing Home, Villa di Teolo (Padova), Italy; 7 Affective Disorders Unit, Casa Di Cura Parco dei Tigli, via Monticello 1, Villa di Teolo, 35037, Padova, Italy

**Keywords:** Suicide, Schizophrenia, Observational study

## Abstract

**Background:**

To identify risk factors for suicide using data from a large, 3-year, multinational follow-up study of schizophrenia (SOHO study).

**Methods:**

Baseline characteristics of 8,871 adult patients with schizophrenia were included in a logistic regression post-hoc analysis comparing patients who attempted and/or committed suicide during the study with those who did not.

**Results:**

384 (4.3%) patients attempted or committed suicide. Completed suicides were 27 (0.3%). The significant risk factors for suicide behaviors were previous suicidality, depressive symptoms, prolactin-related adverse events, male gender and history of hospitalization for schizophrenia.

**Conclusions:**

In view of the observational design of the study and the post-hoc nature of the analysis, the identified risk factors should be confirmed by ad-hoc specifically designed studies.

## Background

One of the most important causes of death among patients with schizophrenia is suicide [1]. It has been established that the risk of suicide is more than 8 times higher among patients with schizophrenia than in the general population [2]. About 20 to 40% of patients with schizophrenia make suicide attempts in their lifetime and around 5% die by their own hand [3]. The lifetime suicide attempt rate in schizophrenia is lower than that in major depressive disorder [3], but attempts are more dangerous, resulting in physical harm significantly more often (44% vs 16%) [4]. Successful lifetime suicide rates in schizophrenia seem to vary considerably from country to country: they have been reported to be approx 20–30:100,000 in Denmark [5] versus 67:100,000 in China [6].

A major hurdle in the prevention of suicide in schizophrenia is the difficulty in evaluating the risk of suicidal behaviors, as suicide in this patient population usually is the result of a sudden, impulsive act that makes the traditional assessment methods based on rating scales and interviews of little use [7].

The current approach to the issue of suicide in schizophrenia rests on antipsychotic treatment, preferentially clozapine, in view of the efficacy it has proved to possess in controlling suicidal behavior in the International Suicide Prevention Trial (InterSePT) trial [8], and on the identification of risk factors and the implementation of preventive measures in high risk patients. At present, there is a general consensus that youth (adolescents and young adults), male gender, Caucasian race, unmarried status, good premorbid function, post-psychotic depression and history of substance abuse and/or suicide attempts are risk factors for suicide behaviors in patients with schizophrenia [1].

The issue of the importance of the endocrine-related adverse events of antipyschotic treatment has recently been raised. Elevation in serum prolactin levels may be associated with distressing adverse events, such as sexual dysfunction, amenorrhoea and galactorrhoea [9].

It is believed that such adverse events may influence adherence to antipsychotic treatment, which is notoriously low in schizophrenia [10,11].

The Schizophrenia Outpatient Health Outcomes (SOHO) study was a prospective, observational study conducted in 10 European countries, which included more than 10,000 outpatients who were initiating or changing antipsychotic medication for the treatment of schizophrenia [12-15].

### Aim of the study

To identify risk factors for suicide behavior, we decided to compare the baseline characteristics of the patients who attempted or committed suicide over a 3-year follow-up in the SOHO study with those patients who did not.

## Methods

These analyses are based on the SOHO study data, a three-year, prospective, observational study of the health outcomes of patients with schizophrenia. A total of 10,972 adult outpatients with a diagnosis of schizophrenia were recruited by psychiatrists in 10 European countries (Denmark, France, Germany, Greece, Ireland, Italy, The Netherlands, Portugal, Spain, UK) from 1 September 2000 to 31 December 2001. Inclusion criteria were: initiating or changing antipsychotic medication for the treatment of schizophrenia; presenting within the normal course of care in the out-patient setting or in the hospital when admission was planned for the initiation of antipsychotic medication and discharge planned within 2 weeks; at least 18 years of age; and not participating in an interventional study. Patients were included regardless of the reason for treatment change (e.g. lack of response, side-effects, etc.) and regardless of whether an antipsychotic drug was being initiated as a replacement for a previous medication, was an addition to existing treatment, or was being initiated for the first time or after a period of no treatment. The study was designed to provide two patient cohorts of approximately equal size: i) patients who initiated therapy with or changed to Olz; and ii) patients who initiated therapy with or changed to a non-Olz antipsychotic. To achieve approximately equal numbers in the Olz and non-Olz groups, different sample fractions entered each cohort. This resulted in a stratified sample, with the Olz group as the over-sampled stratum. In most countries, enrolment was conducted in a systematic alternating order; the first patient was recruited into the Olz cohort, the second patient into the non-Olz cohort, etc. Effort was made to avoid interference with clinical practice. Investigators were instructed to make treatment decisions independent of the study and then evaluate whether patients were eligible for inclusion based on entry criteria and the alternating structure of enrolment; recruitment period was purposely very long and no minimum number of cases was required by investigator.

The study was observational, so the protocol did not provide any instructions regarding treatment or patient management. Outcome data were collected 3 months after baseline and thereafter every 6 months for 3 years.

The SOHO study was approved in all countries either at the site, regional or national level, depending on the country’s regulations and participating sites in each of the countries. All patients gave at least oral informed consent [13].

The baseline data collected included demographic and social information, medical and psychiatric history, body mass index (BMI), severity of symptoms rated using the Clinical Global Impression-Schizophrenia scale dimensions (CGI-SCH), namely overall, positive, negative, depressive and cognitive symptom scores [16,17]. The CGI-SCH scale was physician rated with values ranging from 1 (not ill) to 7 (among the most severely ill patients). Number of suicide attempts from the previous data collection was recorded by the investigator by answering the question: How many times has the patient attempted suicide since the last data collection. Completed suicides were collected in the patient discontinuation form.

Patients with no missing data related to history of suicide attempts and known medications at baseline and at least one post-baseline visit were included in this analysis.

The comparison was made between the patients who, over the 3-year follow-up, had attempted suicide at least once or had committed suicide and those who had never made a suicide attempt. Baseline characteristics were analyzed descriptively. A comparison between patients who attempted suicide and those who committed suicide and between patients included in the analysis and those not was also performed. A logistic regression model for the outcome suicide attempt over follow-up was conducted, including the following baseline covariates as independent variables: sex, age, age at first treatment contact for schizophrenia, CGI-SCH dimensions (positive, negative, cognitive, depressive, overall), hospital admission previous to baseline, body mass index (BMI), history of suicide attempts before baseline (none, one, two or more), suicide attempts in the 6 months before baseline (none, one, two or more), paid work, social contacts in the previous 4 weeks, concomitant medications, presence of extrapyramidal symptoms (EPS), tardive dystonia (TD), sexual-related adverse events and adherence to medications.

The rationale, methods and recruitment of the SOHO study have been described in further detail elsewhere [13], as well as its 6-month, 1-year and 3-years findings [14,15,18].

## Results

Out of the 10,972 patients who were recruited (10,218 with available data), a total of 8,871 patients (86.9%) were included in this analysis. 2,505 of these patients dropped out of the study over 3 years: 8,115 completed one year of observation, 7,271 two years and 6,366 three years of observation. The analysis of patients included in the analysis and those not included showed that those excluded had a higher frequency of lifetime suicide attempts (32% in the patients excluded from the analysis versus 25% in those included) and a history of hospitalization in the previous 6 months before the study (51% in the patients not included in the analysis versus 31% in those included).

A total of 384 (4.3%) patients attempted at least once or committed suicide in the 3 years of the SOHO study. Completed suicides were 27 (0.3%). Ninety-eight patients dropped out of the study after a suicide attempt (1.1%). Most of these patients attempted suicide only once (n = 262 - 3.0%) or twice (n = 50 - 0.6%). The suicide attempt rate was stable throughout the first 2.5 years (0.9-1.0%) and then diminished slightly in the last six months (0.8%).

### Descriptive analysis

There were no significant differences between patients with and without suicide behaviors and in terms of age, gender, occupational status, age at first treatment contact for schizophrenia, time since onset and social activities in the last 4 weeks (Table 1).

**Table 1:** Demography, medical history, baseline data and adverse events by suicide behaviors (attempts or completed suicide)

**Variable**	**Patients without behaviors (n = 8487)**	**Patients with behavors (n = 384)**
***Demographic data and medical history***		
Age (years) mean ± SD	40.1 ± 13.1	38.6 ± 11.8
Male gender (%pat)	57.8%	59.1%
Employment (% pat)	19.7%	17.0%
Social contacts in the previous 4 weeks (% pat)	32.5%	36.8%
Time since first contact (years) mean ± SD	11.4 ± 11.0	11.2 ± 10.2
Age at first contact (years) mean ± SD	28.8 ± 10.6	27.4 ± 10.0
History of attempts (% pat)	23.5%	63.0%
Attempts in the last 6 months (% pat)	3.8%	22.7%
Hospitalizations due to schizophrenia (% pat)	30.7%	48.0%
Concomitant treatment (% pat)	19.0%	20.6%
Anticholinergics	18.1%	31.0%
Antidepressants	36.4%	49.0%
Anxiolyticis/hypnotics	9.8%	14.1%
Mood stabilizer		
Baseline characteristics* (mean ± SD)		
Overall symptom score	4.4 ± 1.0	4.5 ± 1.0
Positive symptom score	3.8 ± 1.4	3.9 ± 1.4
Negative symptom score	4.1 ± 1.3	4.2 ± 1.3
Depressive symptom score	3.4 ± 1.3	3.9 ± 1.4
Cognitive symptom score	3.8 ± 1.3	3.9 ± 1.3
BMI	26.2 ± 4.9	26.0 ± 4.8
***Adverse events (% pat)***		
Extrapyramidal symptoms	37.4%	41.7%
Tardive dyskinesia	9.1%	12.4%
Loss of libido	46.0%	50.1%
Impotence	34.1%	40.3%
Amenorrohea	10.5%	17.2%
Galactorrohea	1.9%	5.0%
Gynecomastia	2.9%	5.8%

BMI body mass index; Pat patients.

* These data are expressed as mean ± SD and represent CGI-SCH scores.

A history of suicidal attempts and recent attempts in the last 6 months were much more common among patients with suicide behaviors: nearly three times as many had attempted suicide in the past and nearly 7 times as many had made their attempts in the last 6 months. Also, a history of hospitalizations on account of schizophrenia was more common amongst patients with suicide behaviors (+56.4%). Depressive symptoms were more severe among patients with suicide behaviors (on average +0.5 points equivalent to a mean increase by 14.7%). Patients who attempted or committed suicide were more likely to be taking antidepressants, anxiolytics/hypnotics and/or a mood stabilizer (Table 1).

The prevalence of adverse events was consistently higher in patients with suicide behaviors compared to patients without (Table 1). The phenomenon was particularly marked for prolactin-related adverse events, namely amenorrhea (+64%), galactorrhea (+163%) and gynecomastia (+200%).

The results are similar when excluding patients who completed suicide from the analysis. The comparison of the patients who completed suicide versus those who did not was limited by the low number of cases. The only statistically significant difference is a higher proportion of males (81% versus 58%).

### Logistic regression

The logistic regression model for the outcome suicide behaviors (attempt or completed suicide) over the follow-up period identified a number of significant risk factors for suicide behaviors (Table 2): a lifetime history of suicide, suicide attempts in the last 6 months, prolactin-related adverse events, male gender, history of hospitalization for schizophrenia, CGI depression score. Age (years) and antipsychotic treatment adherence were not risk factors for suicide attempts. Most of the patients who presented prolactin-related adverse events were female (84% of the patients with those adverse events were female versus 36% in the rest of the sample). There was no significant interaction between prolactin-related adverse events and gender, as regards suicide attempts. The results are similar when excluding patients who completed suicide from the analysis. A further sensitivity analysis was conducted by also including in the analysis the patients with unknown treatment at the baseline visit and those with missing information about suicide attempts at baseline. In the latter case, we have imputed a “0”, the most frequent value, as number of suicide attempts. The results from this model were very similar to those described here.

**Table 2:** Logistic regression model for factors associated to suicide behaviors (attempts or completed suicide) over the follow-up period

	**OR (95% CI)**	**p value**
Age	0.995 (0.985, 1.005)	0.3418
Sex, male	1.614 (1.203, 2.166)	0.0014
CGI-SCH depressive symptoms	1.158 (1.058, 1.268)	0.0015
Hospital admission for schizophrenia	1.38 (1.08, 1.763)	0.0099
Suicide attempts in previous 6 months (1 versus 0)	2.083 (1.462, 2.968)	<.0001
Suicide attempts in previous 6 months (2 or more versus 0)	3.719 (1.852, 7.466)	0.0002
Suicide attempts ever (1 versus 0)	2.35 (1.696, 3.255)	<.0001
Suicide attempts ever (2 or more versus 0)	5.223 (3.913, 6.971)	<.0001
Prolactin-related side effects	2.019 (1.391, 2.929)	0.0002
Complies with medication (always versus never)	0.823 (0.496, 1.366)	0.4514

*CGI-SCH*: Clinical Global Impression-Schizophrenia Scale.

## Discussion

The comparison of the characteristics of patients with schizophrenia with and without suicide behaviors in this large observational study, which included more than 10,000 patients in 10 European countries, has identified a number of significant risk factors for suicide behaviors (attempts or completed suicide): history of suicide attempts, prolactin-related adverse events, male gender, history of hospitalization for schizophrenia, indicated both by CGI depression score. Age and compliance to treatment, on the contrary, did not result to be risk factors for suicide.

These results are consistent with previous studies on the subject, which have already identified history of suicide attempts, male gender and depression as risk factors for suicide [1]. The only exception is age, as previous studies identified young age as a risk factor, whereas age in general did not result to be involved in this study. The comparison of the patients who completed suicide with those who did not was limited given the low number of cases. However, male gender was the only significant difference between those who made a suicide attempt and those who completed suicide.

To our knowledge, this is the first time that prolactin-related adverse events (gynecomastia, galactorrhrea, amenorrhea) are included amongst the risk factors for suicide. Depression, anxiety and hostility have been repeatedly reported to be more frequent in women with hyperprolactinemia. Fava et al. [16] found that women with hyperprolactinemic amenorrhea provided higher self-ratings of these symptoms than both, women with amenorrhea who had normal levels of prolactin and women with regular menstrual cycles. Buckman [19] reported similar mood symptoms in response to elevated prolactin in healthy women. Kellner et al. [20] reported that depression, anxiety and hostility scores in hyperprolactinemic women were similar to those of psychiatric patients and suggested that prolactin induces dysphoric states in its own right. However, the direct impact of increased prolactin on mood and behavior in men is still unclear.

Four relevant limitations should be highlighted in this study. First, this is a post-hoc analysis of the SOHO patient data which included patients who were changed antipsychotic treatment for clinical reasons and are thus not representative of all patients with schizophrenia; moreover, type of antipsychotic medication was not included in the analysis Second, we have not measured prolactin levels, but in SOHO we have assessed adverse events which are potentially related to elevated prolactin levels. Additionally, sexually related adverse events may have multiple causes [21]. Third, suicidal behavior is based on the psychiatrist report using one single question which may be variable and have low reliability. However, we think this bias will be non differential and can rarely create spurious relationships as the ones reported. Finally, patients not included in the analysis because had missing data or were lost to follow-up had somewhat higher frequency of lifetime suicide attempts.

## Conclusions

This study has provided further support to known risk factors for suicide in patients with schizophrenia, such as history of suicide attempts, male gender and depression, and has identified a novel risk factor: prolactin-related adverse events. Further investigations are warranted to clarify the relationship between prolactine related adverse events and risk of suicide.

## Competing interest

The SOHO study was fully sponsored by Eli Lilly & Co.

D. Novick, A. Rossi and S. Frediani are full time employees at Eli Lilly & Co.

J.M. Haro received economic compensation for his participation in the Schizophrenia Outpatient Health Outcomes Advisory Board.

M. Bortolomasi, G. Brugnoli do not have any conflicts of interest to declare.

## Authors’ contributions

D. Novick was the study coordinator and supervised all the aspects of the SOHO study, from study design to data analysis. J.M. Haro supervised and performed all methodological and statistical aspects of the SOHO study, from study design to data analysis. A. Rossi coordinated activities needed for planning and performing analyses included in this article, interpreted results and drafted this article. M. Bortolomasi, G. Brugnoli and S. Frediani contributed to the determination of statistical analyses and contributed to clinical evaluation of statistical analyses, while G. Borgherini formulated the study hypothesis for this analysis and contributed to the clinical evaluation of results. All authors read and approved the final manuscript.

## Significant outcomes

The rate of patients who attempted or committed suicide in a 3-year observational study on 8,871 evaluable outpatients with schizophrenia in 10 European countries was fairly low: 4.3%. Completed suicides were 0.3%.

The significant risk factors for suicide behaviors that were identified were: history of suicide attempts, male gender, depressive symptoms, history of hospitalization for schizophrenia and prolactin-related adverse events.

To our knowledge, this is the first study to identify prolactin-related adverse events, namely amenorrhea, galactorrhea and gynecomastia, as risk factors for suicide in schizophrenia. Additional investigations are needed to confirm that these are indeed.

## Limitations

The results derive from a post-hoc analysis of data collected during an observational trial not designed to specifically evaluate factors predicting suicide.

Prolactine levels were not measured.

The study only included patients who initiated or changed antipsychotic medication treatment during outpatient treatment. Type of antipsychotic medication was not included in the analysis.

Suicidal behavior is based on the psychiatrist report using one single question which may be variable and have low reliability.

Patients not included in the analysis because had missing data or were lost to follow-up, had somewhat higher frequency of lifetime suicide attempts.

## Pre-publication history

The pre-publication history for this paper can be accessed here:

http://www.biomedcentral.com/1471-244X/12/83/prepub
